# Exploring Novel Functions of the Small GTPase Ypt1p under Heat-Shock by Characterizing a Temperature-Sensitive Mutant Yeast Strain, *ypt1-G80D*

**DOI:** 10.3390/ijms20010132

**Published:** 2019-01-01

**Authors:** Chang Ho Kang, Joung Hun Park, Eun Seon Lee, Seol Ki Paeng, Ho Byoung Chae, Yong Hun Chi, Sang Yeol Lee

**Affiliations:** Division of Applied Life Sciences (BK21+) and Plant Molecular Biology and Biotechnology Research Center, Gyeongsang National University, Jinju 52828, Korea; jacobgnu69@gnu.ac.kr (C.H.K.); jazzc@nate.com (J.H.P.); dmstjsl88@hanmail.net (E.S.L.); skpaeng@gmail.com (S.K.P.); truedaisy@hanmail.net (H.B.C.); gandhi37@gnu.ac.kr (Y.H.C.)

**Keywords:** small GTPase, heat-shock, molecular chaperone, structural change, functional switch

## Abstract

In our previous study, we found that Ypt1p, a Rab family small GTPase protein, exhibits a stress-driven structural and functional switch from a GTPase to a molecular chaperone, and mediates thermo tolerance in *Saccharomyces cerevisiae*. In the current study, we focused on the temperature-sensitive *ypt1-G80D* mutant, and found that the mutant cells are highly sensitive to heat-shock, due to a deficiency in the chaperone function of Ypt1p^G80D^. This defect results from an inability of the protein to form high molecular weight polymers, even though it retains almost normal GTPase function. The heat-stress sensitivity of *ypt1-G80D* cells was partially recovered by treatment with 4-phenylbutyric acid, a chemical chaperone. These findings indicate that loss of the chaperone function of Ypt1p^G80D^ underlies the heat sensitivity of *ypt1-G80D* cells. We also compared the proteomes of *YPT1* (wild-type) and *ypt1-G80D* cells to investigate Ypt1p-controlled proteins under heat-stress conditions. Our findings suggest that Ypt1p controls an abundance of proteins involved in metabolism, protein synthesis, cellular energy generation, stress response, and DNA regulation. Finally, we suggest that Ypt1p essentially regulates fundamental cellular processes under heat-stress conditions by acting as a molecular chaperone.

## 1. Introduction

Ypt1p is a member of the Rab family of small GTPases that cycles between an active GTP-bound form and an inactive GDP-bound form. Ypt1p requires guanine nucleotide exchange factor for the GDP-GTP exchange and subsequent activation of the signaling process. In addition, GTPase-activating proteins are also required for hydrolysis of the bound GTP. A large number of small GTPases belonging to the Rab family play a role in vesicular trafficking, and Ypt1p is essential for multiple steps of the yeast secretory pathway, including endoplasmic reticulum to cis-Golgi and cis- to medial-Golgi transport [1]. Although knockout of the *YPT1* gene in yeast is lethal [2], several conditional *ypt1* mutants have been generated and characterized, including GTPase-deficient (*ypt1-S22N* and *ypt1-Q67L*), temperature-sensitive (*ypt1-G80D*), and vesicle transport-defective (*ypt1-1*, *ypt1-2*, and *ypt1-3*) mutants [2,3,4,5,6,7,8]. Hydrolysis of Ypt1p-bound GTP is an essential step in vesicle transport and cell growth, yet the GTPase-deficient *ypt1-Q67L* mutant exhibits no observable defects in protein transport, secretion, membrane morphology, or cell growth at temperatures ranging from 14 °C to 37 °C [4]. Based on these findings, it was concluded that, contrary to the general concept of Ypt1p/Rab function, the GTPase activity of Ypt1p is not essential for Ypt1p-mediated vesicle transport or membrane fusion, or for growth at an elevated temperature (37 °C). On the other hand, the Ypt1p^G80D^ protein has normal GTPase function and the *ypt1-G80D* mutant strain displays normal growth and nearly normal endoplasmic reticulum-to-Golgi vesicle trafficking at typical growth temperature (30 °C), but experiences growth retardation at an elevated temperature (37 °C) [7]. This finding implies that the GTPase activity of Ypt1p is not essential for the growth of yeast at elevated temperatures.

To explain the temperature-sensitive growth phenotype of the *ypt1-G80D* strain, we hypothesized that (a) Ypt1p has a novel function in addition to its well-known GTPase function, (b) the unknown function of Ypt1p is temperature-dependent, and (c) the unknown function of Ypt1p promotes the survival and growth of cells under heat-stress. Accordingly, in our previous study [9], we found that Ypt1p functions as both a GTPase and a molecular chaperone. Furthermore, we found that heat-shock induces a functional switch in Ypt1p from that of a GTPase to that of a molecular chaperone, and this change is driven by a structural switch from a low molecular weight (LMW) to a high molecular weight (HMW) form. In the current study, we examined the conditional chaperone activity of Ypt1p^G80D^ and compared the biochemical properties of the Ypt1p and Ypt1p^G80D^ proteins in vitro and in vivo. Briefly, we found that Ypt1p can switch both structurally and functionally from a GTPase to a molecular chaperone at high temperatures, validating our first two hypotheses. Unlike the wild-type protein, Ypt1p^G80D^ is unable to perform this switch at high temperatures. To gain insight into the cellular processes controlled by Ypt1p under heat-stress conditions, we compared the proteomes of *YPT1* (wild-type) and *ypt1-G80D* mutant cells. Finally, we validated our third hypothesis by showing that the chaperone function of Ypt1p enhances the resistance of cells to heat-stress, while loss of this function in *ypt1-G80D* cells causes a heat-sensitive phenotype.

## 2. Results

### 2.1. Mutant ypt1-G80D Yeast Cells Are Sensitive to Heat-Shock

**Figure 1:**
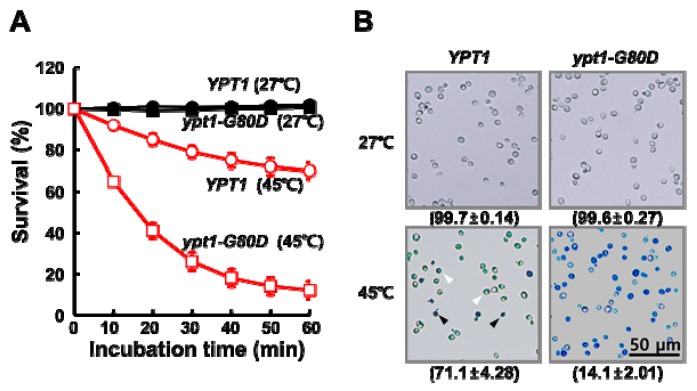
Mutant *ypt1-G80D* Yeast Cells are Sensitive to Heat-Stress. (**A**) Effect of heat treatment on cell viability. *YPT1* and *ypt1-G80D* cells (5 × 10^7^ cells/mL) grown in YPD medium were incubated at 27 °C or 45 °C, and samples were withdrawn at the indicated times for measurement of viable counts. Cell survival (percent) at each time point was calculated as 100× the ratio of the viable count at that time to the viable count at time zero. Error bars: means ± SD of at least 3 independent experiments. (**B**) Trypan blue (TB) exclusion assay of heat-shock-induced cell death. Samples of the *YPT1* and *ypt1-G80D* cells at the 60 min time point in (**A**) were visualized by fluorescence microscopy after staining with TB. The percentages of TB-negative cells are shown below the images. Data are represented as the mean ± SD of at least three independent experiments. White and black arrowheads show examples of TB-negative and -positive cells, respectively. Scale bar, 50 μm.

Isogenic *ypt1* mutant strains grow more slowly than wild-type cells at non-permissive temperatures (e.g., 37 °C) [7], and loss of the chaperone activity of Ypt1p leads to a physiological susceptibility of organisms to heat-shock [10]. To confirm the physiological significance of the G80D mutation in Ypt1p, we compared the viabilities of heat-shocked *YPT1* and *ypt1-G80D* cells (Figure 1). Cultures of the two strains in the mid-exponential growth phase were adjusted to equal cell densities, and aliquots were incubated at 27 °C or 45 °C. Viable counts were determined at regular time intervals (Figure 1A). There was no significant difference in percent survival between the *YPT1* and *ypt1-G80D* strains upon incubation at 27 °C for up to 60 min. However, upon incubation at 45 °C, the percent survival of the *ypt1-G80D* strain was reduced more dramatically than that of the *YPT1* strain (26 ± 4.37% versus 79 ± 2.99% at 30 min, respectively; 12 ± 4.31% versus 70 ± 3.95% at 60 min, respectively). To confirm these results, we assessed cell death using a trypan blue (TB) exclusion assay [11]. The TB exclusion test is based on the principle that only dead cells stain blue because they cannot exclude the dye. As shown in Figure 1B, no TB-positive *YPT1* or *ypt1-G80D* cells were observed after a 60 min incubation at 27 °C. By contrast, after a 60 min incubation at 45 °C, most of the *ypt1-G80D* cells were stained intensely blue, indicating that they were dead, whereas most of the *YPT1* cells were stained only mildly, indicating that they were alive.

### 2.2. Heat-Shock Induces Cytosolic Protein Aggregation in ypt1-G80D Yeast Cells

**Figure 2:**
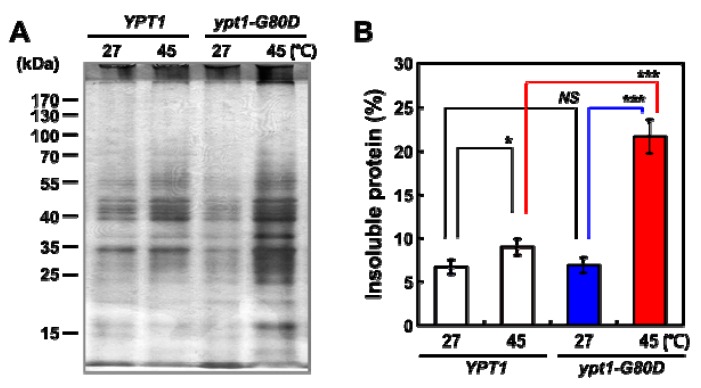
Heat-Shock-Induced Cytosolic Protein Aggregation in Yeast Cells. (**A**) Heat-shock-induced cytosolic protein aggregation in *YPT1* and *ypt1-G80D* cells. The cells (5 × 10^7^ cells/mL) were grown in YPD medium and incubated at 27 °C or 45 °C. Samples were withdrawn at the 60 min time point, and the amounts of insoluble cytosolic protein were determined. The insoluble fractions were subjected to SDS-PAGE followed by silver-staining. Each lane represents the insoluble fraction of 3 × 10^6^ cells. (**B**) Proportions of insoluble fractions in the total cytosolic proteins extracted in (**A**). Red line indicates the comparison between *YPT1* and *ypt1-G80D* samples at 45 °C, blue line shows the comparison of two *ypt1-G80D* samples at 27 °C and 45 °C, whereas black lines represents the comparison of two *YPT1* samples at 27 °C and 45 °C or the comparison between *YPT1* and *ypt1-G80D* samples at 27 °C. Error bars: means ± SD of at least 3 independent experiments. * *p* < 0.05, *** *p* < 0.001, and *NS*, no significance, according to a 2-tailed Student’s *t* test.

Loss of cell viability may be related to the aggregation of thermolabile proteins within cells [12]. Therefore, we compared the abundances of cytosolic protein aggregates in *YPT1* and *ypt1-G80D* cells after incubation at 27 °C or 45 °C for 60 min, using a protocol developed by Tomoyasu et al. [12] that minimizes background signals and enhances the sensitivity of aggregate detection. In *YPT1* cells, the amount of insoluble protein aggregates was only marginally higher at 45 °C than at 25 °C; however, a much larger increase was observed upon incubation at 45 °C in the *ypt1-G80D* cells (Figure 2A). The amount of insoluble protein was quantified by comparing it with that of total protein (Figure 2B). There was no significant difference between the percentages of insoluble protein in *YPT1* and *ypt1-G80D* strains following incubation at 27 °C for up to 60 min. Upon incubation at 45 °C, the percentage of insoluble protein in the *ypt1-G80D* strain was significantly higher than that in the *YPT1* strain (21.5% versus 9.1%, respectively).

### 2.3. Ypt1p^G80D^ does not Undergo a Heat-Shock-Induced Structural Change In Vivo

**Figure 3:**
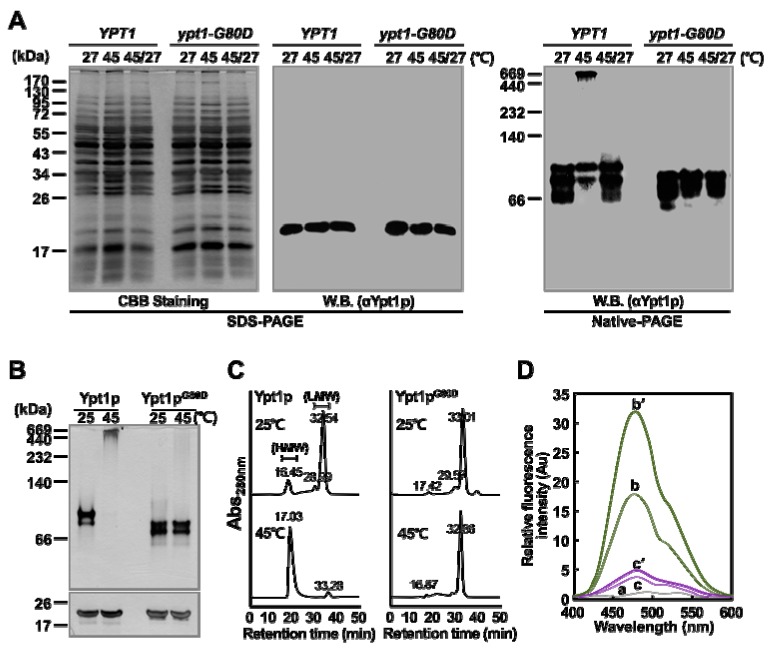
Heat-Shock Induces Changes in the Molecular State of Ypt1p but not Ypt1p^G80D^. (**A**) Changes in the molecular state of Ypt1p and Ypt1p^G80D^ in vivo. *YPT1* and *ypt1-G80D* cells were grown in YPD medium (1 × 10^8^ cells/mL) and incubated at 27 °C or 45 °C for 45 min. Half of the heat-treated cells were transferred to an equal volume of fresh YPD medium containing 100 μg/mL cycloheximide and allowed to recover for 10 h at 27 °C (45/27). Total protein extracts (10 μg) were analyzed by SDS-PAGE, and proteins were visualized by CBB staining (left image). In addition, total protein extracts (50 μg) were analyzed by immunoblotting with a polyclonal anti-Ypt1p antibody after fractionation by SDS-PAGE (middle image) or native-PAGE (right image). W.B., western blotting. (**B**,**C**) Changes in the molecular state of Ypt1p and Ypt1p^G80D^ in vitro. (**B**) Purified bacterially expressed Ypt1p and Ypt1p^G80D^ (3 μg/μL) were incubated at 25 °C or 45 °C for 30 min, subjected to native-PAGE (upper image) or SDS-PAGE (lower image), and then silver-stained. (**C**) SEC analysis of the protein solutions described in (**B**). (**D**) A bis-ANS binding assay to identify heat-shock-induced exposure of hydrophobic domains in Ypt1p and Ypt1p^G80D^. Fluorescence spectra of bis-ANS were measured with excitation at 380 nm and emission scanning at 400–600 nm. The samples used were 10 μM bis-ANS (a), 10 μM bis-ANS plus 30 μM Ypt1p incubated at 25 °C (b) or 45 °C (b′), and 10 μM bis-ANS plus 30 μM Ypt1p^G80D^ incubated at 25 °C (c) or 45 °C (c′) for 20 min.

In our previous research, we found that Ypt1p undergoes reversible temperature-dependent structural changes [9]. Therefore, we investigated whether Ypt1p^G80D^ also undergoes temperature-dependent structural modifications in vivo and in vitro. First, *YPT1* and *ypt1-G80D* cells were subjected to heat-shock at 45 °C for 45 min, and then half of the cells were allowed to recover at 27 °C for 10 h in fresh growth medium containing the protein synthesis inhibitor cycloheximide. Total protein extracts of these samples were fractionated by SDS-PAGE and native-PAGE, and Ypt1p/Ypt1p^G80D^ was detected by immunoblotting with an anti-Ypt1p polyclonal antibody prepared in our laboratory [9]. As expected, a single protein band of 23.5 kDa, corresponding to the size of monomeric Ypt1p/Ypt1p^G80D^, was detected in all samples on the SDS-PAGE gel (Figure 3A). The native-PAGE analysis confirmed that heat-shock treatment induced the reversible formation of HMW protein complexes containing Ypt1p in vivo, as reported previously [9]. By contrast, in the *ypt1-G80D* cell extract, the antibody detected a relatively narrow range of band sizes. Notably, HMW Ypt1p^G80D^ complexes were not observed in extracts of heat-treated *ypt1-G80D* cells (Figure 3A). Interestingly, the LMW bands of Ypt1p/Ypt1p^G80D^ show different patterns in the *YPT1* and *ypt1-G80D* cells, with doublets at different positions on native PAGE (Figure 3A). This was similar for the recombinant proteins analyzed on native PAGE (Figure 3B, upper gel). Considering that Glycine, the 80th residue of Ypt1p is replaced with Aspartic acid in Ypt1p^G80D^, the amino acid replacement could affect size shifts on the gel by affecting secondary or tertiary structure of the protein [13,14].

The inability of *ypt1-G80D* cells to form heat-induced HMW aggregates containing Ypt1p^G80D^ was confirmed by size exclusion chromatography (SEC). Total protein extracts from heat-treated and untreated *ypt1-G80D* cells were fractionated by SEC (Figure S1). The fractions were analyzed by SDS-PAGE, and the Ypt1p^G80D^ contents were determined by western blotting (Figure S1). In *ypt1-G80D* cells, Ypt1p^G80D^ was mainly detected in LMW protein fractions (≤ 140 kDa), regardless of heat treatment.

### 2.4. Ypt1p^G80D^ does not Undergo a Heat-Shock-Induced Structural Change In Vitro

Next, we investigated the possibility of heat-induced polymerization of purified bacterially expressed recombinant Ypt1p^G80D^. As observed in our previous study [9], a heat-induced structural change in purified recombinant Ypt1p was confirmed (Figure 3B,C). Native-PAGE analyses revealed no differences between the protein banding patterns of recombinant Ypt1p^G80D^ following incubation at 25 °C or 45 °C, and most of the Ypt1p^G80D^ protein was detected as a LMW oligomer at both temperatures (Figure 3B). Furthermore, SEC analyses revealed no marked differences between the samples incubated at 25 °C or 45 °C (Figure 3C). In the Ypt1p^G80D^ sample, major protein peaks were observed at retention times of 33.01 min and 32.86 min following incubation at 25 °C and 45 °C, respectively, indicating the formation of LMW oligomers under both conditions. Unlike in the Ypt1p sample, the SEC protein peak with a retention time of approximately 17 min, corresponding to HMW oligomers, was almost undetectable in the Ypt1p^G80D^ samples incubated at 25 °C or 45 °C, confirming that Ypt1p^G80D^ is unable to form HMW oligomers upon heat treatment.

We also examined changes in the hydrophobicities of purified bacterially expressed Ypt1p and Ypt1p^G80D^ upon heat treatment using 4,4′-bis (1-anilinonaphthalene 8-sulfonate) (bis-ANS) as a probe. Binding of Bis-ANS to hydrophobic patches on proteins results in fluorescence with an emission maximum of approximately 470 nm [15]. As shown in our previous study [9], when Ypt1p was incubated with bis-ANS (Figure 3D), the fluorescence peak at 470 nm was larger when the incubation was carried out at 45 °C rather than 25 °C. By contrast, Ypt1p^G80D^ exhibited very little fluorescence at 470 nm when incubated with bis-ANS at 25 °C, and there was no significant increase in this fluorescence when the incubation was carried out at 45 °C (Figure 3D). These results indicate that the conformations of Ypt1p^G80D^ and Ypt1p differ at 25 °C, and the conformation of Ypt1p^G80D^ does not change at 45 °C. Overall, the results show that Gly^80^ is required for both the heat-induced increase in the surface hydrophobicity of Ypt1p (Figure 3D) and the formation of HMW Ypt1p homo-polymers (Figure 3B,C). They also suggest that Ypt1p^G80D^ has a smaller amount of exposed hydrophobic patches than Ypt1p, and subsequently a lower tendency to form HMW complexes.

### 2.5. Ypt1p^G80D^ Retains GTPase Activity but Loses Molecular Chaperone Activity

Since Ypt1p^G80D^ exists as a LMW form regardless of temperature and has a lower hydrophobicity than Ypt1p (Figure 3D), we examined whether the structural difference between Ypt1p and Ypt1p^G80D^ leads to a difference in function. To this end, we examined the chaperone and GTPase activities of Ypt1p and Ypt1p^G80D^.

**Figure 4:**
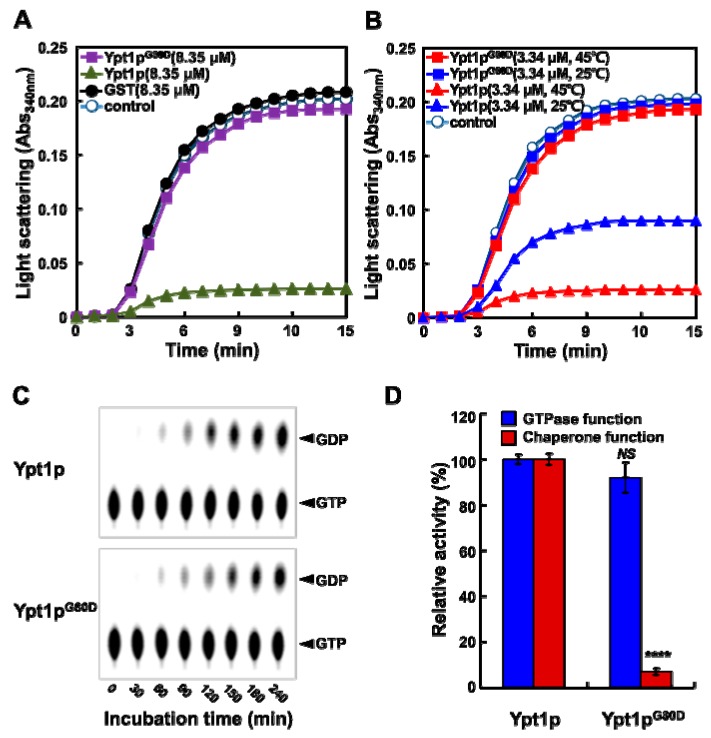
Ypt1p^G80D^ has GTPase Activity but not Molecular Chaperone Activity. (**A**,**B**) Chaperone activity assay. Light scattering was monitored at 340 nm over a 15 min incubation period. Shown are representative data out of at least three independent experiments. (**A**) Solutions of MDH (1.67 μM) alone (**-o-**) or with 8.35 μM GST (**-●-**), Ypt1p (**-▲-**), or Ypt1p^G80D^ (**-■-**) in 50 mM HEPES (pH 8.0) were incubated in a spectrophotometer cell at 45 °C. (**B**) Solutions of MDH (1.67 μM) alone (**-o-**) or with 3.34 μM Ypt1p pretreated at 25 °C (**-▲-**) or 45 °C (**-▲-**) or 3.34 μM Ypt1p^G80D^ pretreated at 25 °C (**-■-**) or 45 °C (**-■-**) in 50 mM HEPES (pH 8.0) were incubated in a spectrophotometer cell at 45 °C. (**C**) GTPase activity assay. Recombinant Ypt1p and Ypt1p^G80D^ (2 μg each) were incubated with [α-^32^P]GTP at 30 °C, and samples of the reaction mixtures were withdrawn at different time points for analysis by TLC. Shown is a representative image out of at least three independent experiments. (**D**) Relative GTPase (240 min) and chaperone (15 min) activities of Ypt1p and Ypt1p^G80D^. The activities of Ypt1p were set to 100%. Data are represented as the mean ± SD of at least three independent experiments. **** *P* < 0.0001, and *NS*, no significance, according to a Student’s *t* test.

To investigate chaperone activity, we measured the abilities of Ypt1p and Ypt1p^G80D^ to prevent heat-induced denaturation of the substrate proteins malate dehydrogenase (MDH) and citrate synthase (CS) [10,16]. The formation of insoluble protein aggregates of denatured MDH and CS was monitored by measuring light scattering at 340 nm (Figure 4A and Figure S2). Light scattering increased rapidly when MDH (1.67 μM) and CS (2 μM) were heated alone or with a 5-fold molar excess (8.35 μM) of Ypt1p^G80D^ or the negative control glutathione-S-transferase (GST) protein. However, when MDH (1.67 μM) and CS (2 μM) were heated in the presence of a 5-fold molar excess of Ypt1p, the aggregation was successively prevented, confirming the molecular chaperone activity of Ypt1p.

Next, we examined the temperature-dependency of the chaperone activities of Ypt1p and Ypt1p^G80D^. Light scattering increased rapidly when MDH (1.67 μM) was heated alone or with a 2-fold molar excess (3.34 μM) of Ypt1p^G80D^ that had previously been incubated for 30 min at 25 °C or 45 °C (Figure 4B). However, when MDH (1.67 μM) was heated in the presence of a 2-fold molar excess of Ypt1p that was incubated at 25 °C previously, the aggregation was reduced by approximately 50%, and was reduced more substantially in the presence of Ypt1p that was incubated at 45 °C previously (Figure 4B). These findings suggest that the chaperone activity of Ypt1p is enhanced by incubation at a high temperature, whereas Ypt1p^G80D^ does not have molecular chaperone function, regardless of temperature.

The GTPase activities of bacterially expressed recombinant Ypt1p and Ypt1p^G80D^ were comparable (Figure 4C). With both proteins, GDP formation was observed after 1 h and approximately 50% of the [α-^32^P]GTP substrate was hydrolyzed within 6 h. Ypt1p^G80D^ retained almost 90% of the GTPase activity of native Ypt1p, but less than 10% of the chaperone activity (Figure 4D). These results suggest that the increased heat-stress sensitivity of *ypt1-G80D* mutants is likely related to the loss of Ypt1p chaperone activity.

### 2.6. PBA Increases the Thermo Tolerance of ypt1-G80D Cells

**Figure 5:**
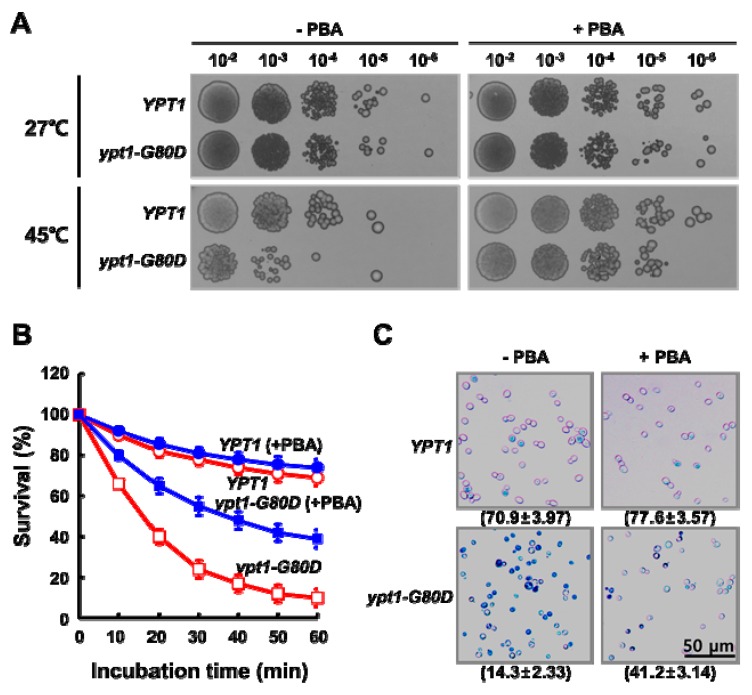
PBA Increases the Thermo Tolerance of *ypt1-G80D* Cells. (**A**) Yeast spot assay. Yeast cells (5 × 7 cells/mL) were grown in YPD medium and incubated at 27 °C or 45 °C for 1 h in the presence (+PBA) or absence (-PBA) of 1 mM PBA. Samples of the *YPT1* and *ypt1-G80D* cells were withdrawn for analysis. Aliquots (6 μL) of 10-fold serial dilutions of these cell suspensions were spotted onto YPD plates, and the plates were photographed after incubation at 27 °C for 3 days. (**B**) Effect of PBA on heat-shock resistance of the *YPT1* and *ypt1-G80D* cells. Yeast cells (5 × 7 cells/mL) were grown in YPD medium and incubated at 45 °C in the presence (+PBA) or absence (-PBA) of 1 mM PBA. Samples were withdrawn at the indicated times for measurement of viable counts. Cell survival (percent) at each time point was calculated as 100× the ratio of the viable count at that time to the viable count at time zero. Error bars: means ± SD of at least 3 independent experiments. (**C**) Trypan blue exclusion assay of heat-shock-induced cell death. Samples of the *YPT1* and *ypt1-G80D* cells at the 60 min time point in (**B**) were visualized by fluorescence microscopy after staining with trypan blue. The percentages of TB-negative cells are shown below the images. Data are represented as the mean ± SD of at least three independent experiments.

To further confirm that the lack of chaperone function in Ypt1p^G80D^ accounts for the thermal sensitivity of the *ypt1-G80D* mutant, we compared the heat sensitivities of *YPT1* and *ypt1-G80D* strains by incubating them at 27 °C or 45 °C for 1 h in the presence or absence of sodium 4-phenylbutyric acid (PBA), a chemical chaperone [17] (Figure 5). At 27 °C, there was no difference between the growth of the strains in either the absence or presence of PBA. Following incubation at 45 °C, the viability of the *ypt1-G80D* mutant was reduced to a greater extent than that of the *YPT1* strain in the absence of PBA. The viabilities of both strains were improved in the presence of PBA, but the improvement was more marked for the *ypt1-G80D* mutant than the *YPT1* strain (Figure 5A). Next, we compared survival of the two strains after incubating comparable concentrations of mid-logarithmic phase cells for up to 60 min at 45 °C in the presence or absence of 1 mM PBA (Figure 5B). PBA had no effect on the viability of *YPT1* cells at 45 °C, as determined by measuring viable counts or using the TB exclusion assay (Figure 5B,C). By contrast, PBA exposure increased the viability of *ypt1-G80D* cells (Figure 5B,C), although it was not restored to the level seen for the *YPT1* strain. These results support the conclusion that the thermal sensitivity of the *ypt1-G80D* mutant is due to a deficiency in the heat-induced chaperone function of Ypt1p^G80D^.

### 2.7. Identification of Putative Ypt1p-Regulatory Proteins under Heat-Shock

**Figure 6:**
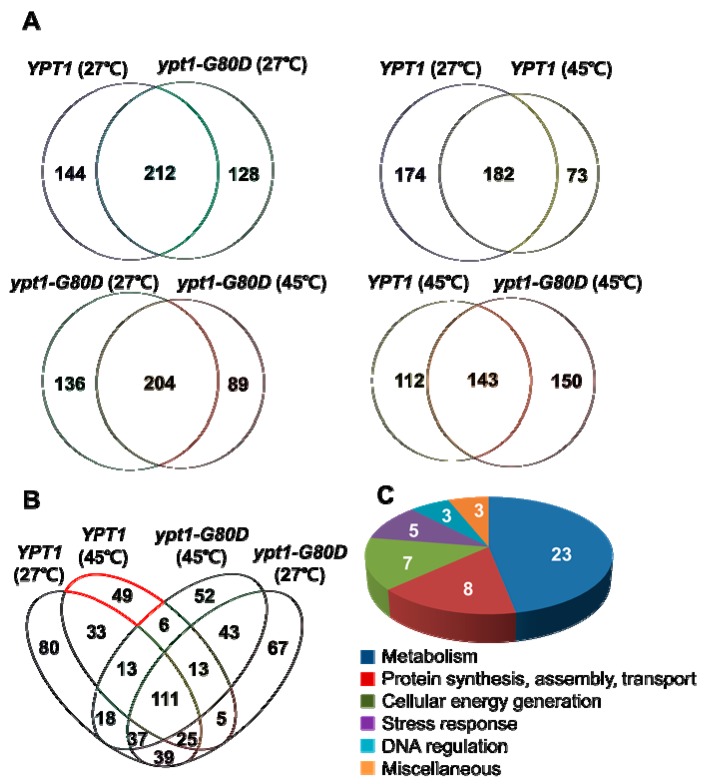
Analyses of LC/MS Data. (**A**) Venn diagrams showing the numbers of proteins identified by the LC/MS analysis in extracts of *YPT1* and *ypt1-G80D* cells incubated at 27 °C (upper left), extracts of *YPT1* cells incubated at 27 °C or 45 °C (upper right), extracts of *ypt1-G80D* cells incubated at 27 °C or 45 °C (lower left), and extracts of *YPT1* and *ypt1-G80D* cells incubated at 45 °C (lower right). (**B**) Venn diagram showing the relationships between the proteins identified by the LC/MS analysis in extracts of *YPT1* and *ypt1-G80D* cells incubated at 27 °C or 45 °C. The group of 49 proteins that was induced at 45 °C in *YPT1* cells but not *ypt1-G80D* cells is indicated by the red outline. (**C**) Pie chart showing distribution of these 49 proteins among different cellular processes.

To identify intracellular Ypt1p-regulatory proteins in heat-shocked yeast cells, *YPT1* and *ypt1-G80D* cells were incubated at 27 °C or 45 °C for 1 h and total proteins were analyzed by tandem MS (MS/MS) using a Velos LTQ mass spectrometer [18] (Figure S3). Trypsinized peptides were separated by reversed-phase nanoflow liquid chromatography (LC) followed by MS/MS sequencing in the high energy collision dissociation (HCD) mode, and the peptide spectra were searched against the Uniprot database (http://www.uniprot.org/) of *Saccharomyces cerevisiae* proteins, using the SEQUEST algorithm. For highest fidelity, the cut-off was set at 99% false discovery rate. From the total protein fraction of *YPT1* cells, 356 and 255 proteins were identified at 27 °C and 45 °C, respectively, whereas the corresponding numbers from *ypt1-G80D* cells were 340 and 293 proteins, respectively. A total of 73 proteins were specifically enriched in *YPT1* cells incubated at 45 °C compared with 27 °C (Figure 6). By comparison, 89 proteins were specifically enriched in *ypt1-G80D* cells incubated at 45 °C compared with 27 °C (Figure 6). Six heat-induced proteins were common to both strains, including alcohol dehydrogenase 1p (Adh1p, E7LZZ9), phosphorylase (Gph1p, B3LKC1), vacuolar protein sorting 29p (Vps29p, E7KDB3), ADP-ribosylation factor 1 (P11076), lipoamide dehydrogenase 1p (Lpd1p, E7NH76), and YMR051C-like protein (E7QCY4). A total of 49 proteins were specifically enriched in heat-treated *YPT1* cells but were not identified among the heat-shock-enriched proteins in *ypt1-G80D* cells, suggesting that these proteins are directly or indirectly dependent on Gly^80^ of Ypt1p (Table 1 and Table S1). Among the *YPT1*-specific heat-shock-induced proteins, 23 were annotated as involved in metabolism; 8 were involved in protein synthesis, assembly, or transport; 7 were involved in cellular energy generation, 5 were involved in stress response, 3 were involved in DNA regulation, and the remaining 3 proteins were annotated as “miscellaneous”. This finding suggests that abrogation of heat-shock-induced Ypt1p chaperone function by the G80D mutation lowers cell viability largely by hindering metabolism and cellular energy generation.

## 3. Discussion

Organisms need to be able to adapt to their environment because environmental nutritional usability, osmotic balance, temperature, and presence of harmful substances are constantly changing. To protect against these external stresses [19,20], all aerobic organisms are equipped with a wide range of protective proteins, including a diverse range of molecular chaperones such as the heat-shock proteins, the small heat-shock proteins, and several redox chaperones [21,22]. Recent reports showed that some plant proteins play a role in cellular protection against heat-stress via the acquisition of new functions endowed by heat-induced structural changes [16,23,24,25]. In addition, two yeast cytosolic peroxiredoxins (Prxs) change from a LMW form to a HMW form following heat-shock or oxidative stress, conferring stress resistance to cells [10]. This structural change is accompanied by functional switching from a peroxidase to a molecular chaperone. Our previous study [9] showed that heat-shock induces a reversible polymerization of Ytp1p into a HMW form in vivo and in vitro, and this structural change abolishes the original GTPase activity but confers a new molecular chaperone activity on the protein. If cells are allowed to recover from heat-stress, Ypt1p reverts back to the LMW form in vivo.

Loss of the function of molecular chaperone genes reduces the ability of cells to protect against environmental stresses [26,27,28]. For example, yeast cells become very sensitive to heat-shock stress following loss of the *cPrxI* and *cPrxII* genes [10]. As expected, mutations in the *YPT1* gene cause defective yeast growth at a non-permissive temperature. Representatively, the *ypt1-G80D* mutant strain shows a temperature-sensitive phenotype [7]; however, the reason for this defect had not previously been identified. In the current study, we examined the physiological effects of the G80D mutation in *ypt1-G80D* mutant cells. We found that Ypt1p^G80D^ retained most of the GTPase activity of Ypt1p but was unable to form HMW complexes and lacked chaperone function (Figure 3, Figure 4, Figures S1 and S2). The *ypt1-G80D* mutant strain experienced a greater loss of viability than the *YPT1* strain when exposed to heat-shock (Figure 1 and Figure 5), suggesting that heat-stress-driven polymerization and switching of Ypt1p function to a molecular chaperone is critical for cell survival. Addition of a chemical chaperone to the growth medium of the *ypt1-G80D* strain reduced its heat sensitivity (Figure 5). Therefore, we suggest that an increase in the proportion of HMW Ypt1p polymers in a cell experiencing heat-stress helps to prevent the stress-induced aggregation of intracellular proteins that leads to loss of viability (Figure 2).

In general, hydrophobic regions are buried inside proteins that are properly folded. However, when cells are exposed to severe stress, such as heat-shock or oxidative stress, the proteins are denatured and the hydrophobic regions are exposed. Subsequently, the external hydrophobic regions are recognized by molecular chaperones, which target a number of denatured proteins [29,30]. Accordingly, we observed an abundance of numerous insoluble proteins in the *ypt1-G80D* strain that lacked the chaperone function of Ypt1p (Figure 2). Proteomic analysis of proteins extracted from unstressed and heat-shocked *YPT1* and *ypt1-G80D* cells identified 49 proteins that were heat-shock-induced in *YPT1* cells but not *ypt1-G80D* cells, implying that the chaperone function of Ypt1p is essential for the increase in abundance of these particular proteins under stress (Figure 6). Included in these 49 proteins were various enzymes involved in glycolysis and the tricarboxylic acid cycle, and proteins necessary for cellular energy generation, protein synthesis, protein assembly, and protein transport (Table 1). These results suggest that loss of Ypt1p chaperone function may cause problems in cell metabolism, protein synthesis, and energy generation under heat-shock. Stress-seventy subfamily A 1p (Ssa1p, E7Q0L2), ATP synthase subunit beta (Atp2p, E7Q5S7), inorganic pyrophosphatase (Ipp1p, P00817), porin (Por1p, B3LNR6), and translation elongation factor 2 (Eft2p, E7NN07) showed the highest numbers of peptide-spectrum matches among the 49 proteins (Table S1), and previous studies found that complete or partial loss of the functions of these proteins by mutation of the corresponding genes increases the heat sensitivity of yeast cells [26,27,28]. The fact that these proteins were less abundant in *ypt1-G80D* cells than *YPT1* cells under heat-shock conditions may explain why *ypt1-G80D* cells are sensitive to this type of stress. Further studies of ypt1 mutants are required to examine this possibility.

Small G-proteins affect most biological processes by controlling complex cell signaling processes. As a result of their extensive cellular roles, inadequate control and functional failure of small G-proteins often lead to human diseases [31]. For example, dysfunction of Rab GTPase contributes to genetic or acquired human diseases [32,33]. Therefore, small G-proteins and their regulators are targets for the development of various human medicines. Previous studies have mainly focused on the functions of small G-proteins as GTPases; however, our current research suggests that it may be possible to find new ways to cure human diseases by expanding the horizon of small G-proteins to a new family of molecular chaperones. In this regard, previous findings that various molecular chaperones are closely related to human diseases should be considered [34,35].

## 4. Materials and Methods

### 4.1. Yeast Strains, Survivability Assays, and TB Exclusion Assay

Isogenic *YPT1* (SVL82; *MaTα*, *ade2*, *his3*, *leu2*, *trp1*, *ura3*, *can1*) and *ypt1-G80D* (SVL422; *MaTα*, *ade2*, *his3*, *leu2*, *trp1*, *ura3*, *can1*, *ypt1-G80D*) *S. cerevisiae* W303 strains were grown in YPD medium at 27 °C [7]. For the viable count assay, yeast cells were grown overnight in YPD medium and cells (5 × 10^7^ cells/mL) in fresh YPD medium were incubated at 27 °C or 45 °C. Samples were withdrawn at 0, 10, 20, 30, 40, 50, and 60 min after incubation, and survival assays were performed. The numbers of viable cells or colony forming units were determined by plating suitable dilutions onto YPD agar plates and counting the colonies that appeared after 2–3 days of incubation at 27 °C.

Samples of the *YPT1* and *ypt1-G80D* cells at the 60 min time point in the survival assay were subjected to TB staining and spot assays. For TB staining, 1 mL of cells was harvested by centrifugation, washed with PBS (140 mM NaCl, 2.7 mM KCl, 10 mM Na_2_HPO_4_, and 1.8 mM KH_2_PO_4_ [pH 7.6]), resuspended in PBS, and stained with 0.4% TB for 5–10 min. The cells were examined under a fluorescence microscope (Olympus Optical Co., Tokyo, Japan), and results were documented using software provided by the company. For spot assays, cultures were adjusted to an Abs_600nm_ of 1.0 and aliquots (6 μL) of 10-fold serial dilutions were spotted onto YPD agar. The plates were examined after incubation at 27 °C for 2–3 days.

### 4.2. Analysis of Heat-Shock-Induced Cytosolic Protein Aggregation in Yeast Cells

Samples of the cell cultures at the 60 min time point in the survival assay underwent measurements of cytosolic protein aggregation, as described previously [10]. Yeast cells harvested at an identical cell density were resuspended in lysis buffer (50 mM potassium phosphate buffer [pH 7.0], 1 mM EDTA, 5% glycerol, and 1 mM PMSF) containing protease inhibitor cocktail (Sigma-Aldrich, St. Louis, MO, USA) and incubated at 30 °C for 30 min after adding 0.25 volumes of zymolase 20T (10 mg/mL). The cells were broken by beating them three times with acid-washed glass beads in a bead beater (Biospec Products, Bartlesville, OK, USA) for 1 min. Each beating was followed by cooling at 4 °C for 2 min. After removing intact cells by brief centrifugation, the mixture was separated into an insoluble pellet fraction and a cytosolic supernatant by centrifugation at 15,000 *g* for 30 min. The insoluble pellet fraction containing the membrane and aggregated proteins was then resuspended in 320 μL of lysis buffer by brief sonification. Membrane proteins were removed by adding 80 μL of 10% (*v*/*v*) NP40 and centrifuging at 15,000 *g* for 20 min. The NP40-insoluble protein aggregates in the pellet were then analyzed by gel electrophoresis. Protein concentrations in the cytosolic and insoluble fractions were measured to analyze the proportions of the insoluble fractions in the total cytosolic proteins.

### 4.3. Construction of Expression Plasmids

The G80D-F (5′-TCTTACTACCGTGATTCGCATGGGATC-3′) and G80D-R (5′-GATCCCATGCGAATCACGGTAGTAAGA-3′) primers were used to generate the G80D mutation. Substitution of single amino acids was performed using the QuickChange™ Site-Directed Mutagenesis Kit (Stratagene, La Jolla, CA, USA), as described previously [36]. To generate pGEX-ypt1^G80D^, mutation was performed by targeting the pGEMT-YPT1 plasmid [9]; the insert was then released by digesting the resulting plasmid with *Bam*HI and *Hind*III, and cloned into the corresponding sites of the pGEX-2T vector.

### 4.4. Purification of Recombinant Proteins and Production of the Polyclonal Antibody

The pGEX-YPT1 and pGEX-ypt1^G80D^ vectors were transformed into *Escherichia coli* BL21(DE3)pLysS cells. The cells were then cultured at 37 °C in LB medium supplemented with ampicillin (50 μg/mL). At an Abs_600nm_ of approximately 0.5–0.6, protein expression was induced by the addition of 0.2 mM isopropyl-β-D-thiogalacto-pyranoside. After an additional 4 h culture at 30 °C, the cells were harvested by centrifugation at 6000 *g* for 6 min. The pellets were resuspended in PBS buffer containing 1 mM PMSF and stored at –70 °C. GST-fused Ypt1p proteins were purified from the cells using GSH-agarose resin, and the GST-tag was removed by thrombin cleavage, as described previously [37]. Ypt1p proteins were further purified using a TSK heparin-5PW HPLC column (7.5 × 75 mm), as described previously [38]. DnaK, a possible co-purifying contaminant on GSH columns, was removed using an ATP-agarose column according to the manufacturer’s instructions (Amersham Pharmacia Biotech, Piscataway, NJ, USA). The pure Ypt1p proteins were dialyzed against 20 mM HEPES (pH 8.0) before use. Purified Ypt1p was used for the immunization of rabbits to obtain a polyclonal antibody.

### 4.5. Size Exclusion Chromatography

SEC was performed on a Superdex 200 HR 10/30 column equilibrated with 50 mM HEPES (pH 8.0) buffer containing 100 mM NaCl, at a flow rate of 0.5 mL/min (AKTAFPLC; Amersham Biosciences, Piscataway, NJ, USA), as described previously [10]. Protein (Abs_280nm_) peaks were pooled and concentrated using Centricon YM-30 (Millipore Corp., Bedford, MA, USA).

### 4.6. Assay of GTPase Activity

A thin layer chromatography (TLC) technique [39] was modified slightly for measurement of GTPase activity. The GTPase reaction was allowed to proceed at 30 °C in 200 μL of HEDL buffer (20 mM Tri-HCl [pH 7.5], 2 mM EDTA, and 10 mM DTT) containing 0.1 μM [α^32^P]GTP and 2 μg of Ypt1p. At appropriate time intervals, 10 μL aliquots were withdrawn and added to 10 μL of 0.5 M EDTA (pH 8.0) to stop the reaction. Subsequently, a 2 μL aliquot of this mixture was spotted onto a PEI-cellulose TLC plate. The plates were developed in 0.5 M KH_2_PO_4_ (pH 3.4), dried, and exposed to X-ray film as described previously [40].

### 4.7. Measurement of bis-ANS Fluorescence

A reaction mixture containing proteins (30 μg/mL in 20 mM HEPES, pH 8.0) and bis-ANS (10 μM; Sigma-Aldrich) was incubated at various temperatures for 30 min, and then the fluorescence spectrum between 400 and 600 nm was obtained at an excitation wavelength of 380 nm using a SFM25 spectrofluorometer (Kontron, Zurich, Switzerland).

### 4.8. LC/MS Analysis

*YPT1* and *ypt1-G80D* cell cultures in YPD medium were adjusted to an Abs_600nm_ of 1.0 and incubated at 27 °C or 45 °C for 1 h. The cells were harvested by centrifugation, and the culture medium was removed completely by pipetting. To prepare total protein samples, yeast cells were lysed in lysis buffer (8 M urea, 100 mM NaH_2_PO_4_, and 50 mM Tris [pH 8.0]) using a pestle and mortar. Subsequently, the 8 M urea samples were diluted with an equal volume of 20 mM Tris [pH 8.0] to a final concentration of 4 M urea. Trypsin was added to the solution in an enzyme/protein ratio of 1:50, along with 2 mM CaCl_2_. After digestion, the tryptic digests were desalted using a C18 solid-phase extraction pipette tip (SPEC PT C18; Varian, Lake Forrest, CA, USA), vacuum-dried, and reconstituted in 10 mL of 95% water, 5% acetonitrile, and 0.1% formic acid. The samples were analyzed by electrospray ionization MS using a system consisting of a nanoflow liquid chromatograph (nanoAcquity; Waters Corp., Milford, MA, USA) connected online to an electrospray ionization FT/ion-trap mass spectrometer (LTQ Orbitrap Velos; Thermo Fisher Scientific, San Jose, CA, USA). LC separation employed a 100 × 365 μm fused silica capillary microcolumn packed with 15 cm of 3 μm diameter, 100 Å pore size, C18 beads (Magic C18; Bruker, Billerica, MA, USA), with the emitter tip pulled to approximately 2 μm using a laser puller (Sutter Instruments). Peptides were loaded onto the column at a flow rate of 500 nL/min for 30 min, and then eluted over 120 min at a flow rate of 200 nL/min with a gradient of 2–30% acetonitrile in 0.1% formic acid. Full mass scans were performed in the FT orbitrap between 300 and 1500 mass-to-charge ratio at a resolution of 60,000, followed by ten MS/MS HCD scans of the ten highest intensity parent ions at 42% relative collision energy and 7500 resolution, with a mass range starting at 100 mass-to-charge ratio. Dynamic exclusion was enabled with a repeat count of two over the duration of 30 s and an exclusion window of 120 s. The acquired precursor MS and MS/MS spectra were searched against the Uniprot database (http://www.uniprot.org/) of *Saccharomyces cerevisiae* proteins using SEQUEST version 1.2 (ThermoFisher Scientific). Masses of the precursor and fragment ions were treated as monoisotopic. The database search allowed for up to two missed trypsin cleavages, and ion masses were matched with a mass tolerance of 10 ppm for precursor masses and 0.1 D for HCD fragments. The data were filtered using a 1% false discovery rate [41], with a minimum of two peptide matches required for confident protein identification. The database search using the peptide sequences from the MS analyses identified 356 and 255 proteins for the 30 °C or 45 °C *YPT1* samples, respectively, and 340 and 293 proteins for the *ypt1-G80D* cells, respectively (Table S1). The identified proteins were further analyzed for shared or specific groups using the free software VENNY [42].

## Figures and Tables

**Table 1:** Categorized Lists of Putative Ypt1p-Regulatory Proteins under Heat-Shock. See Table S1 for further details. “# proteins” means number of proteins in the groups.

Groups (# proteins)	Putative Ypt1p-Regulatory Proteins under Heat-Shock
Metabolism (23)	Inorganic pyrophosphatase, Glucose-6-phosphate isomerase, Ach1p, YKR097Wp-like protein, Thr4p, 6-phosphogluconate dehydrogenase, Alpha-ketoglutarate dehydrogenase, Carboxypeptidase Y inhibitor, Ses1p, YGL202Wp-like protein, Transaldolase, IDH2 (Isocitric dehydrogenase 2), Putative uncharacterized protein (A6ZLW7), Pbi2p, IDP2 (Isocitrate dehydrogenase), Branched chain a.a. aminotransferase, YGR124Wp-like protein, Bts1p, Enolase, Ypr1p, Erg10p, Cys4p, K7_Ade17bp
Protein synthesis, assembly, and transport (8)	Eft2p, Om45p, Ses1p, Tropomyosin-1, YFL037Wp-like protein, KAP123 (Karyopherin beta 4), YBL050Wp-like protein, Rpl7bp
Cellular energy generation (7)	ATP synthase subunit beta, Porin, Rhr2p, ADK1 (Adenylate kinase), Sdh1p, Atp16p, YBR039Wp-like protein, Cytochrome b-c1 complex subunit 7
Stress response (5)	Ssa1p, Ssa3p, Prx1p, SIP18, YML128Cp-like protein
DNA regulation (3)	REV3 (DNA polymerase), YPL016Wp-like protein, Mbp1p
Miscellaneous (3)	Putative uncharacterized protein (A6ZYB0), SOF1 (Conserved protein), Tma19p

## Supplementary Materials

Supplementary materials can be found at http://www.mdpi.com/1422-0067/20/1/132/s1.

## Abbreviations

bis-ANS	4,4′-bis (1-anilinonaphthalene 8-sulfonate)
CBB	Coomassie Brilliant Blue
CS	citrate synthase
GST	glutathione-S-transferase
HMW	high molecular weight
LC-MS	Liquid chromatography-mass spectrometry
LMW	low molecular weight
MDH	malate dehydrogenase
PBA	sodium 4-phenylbutyric acid
Prx	peroxiredoxin
SEC	size exclusion chromatography
TB	trypan blue
TLC	thin layer chromatography
