# Supplementary material for: Exploring Novel Functions of the Small GTPase Ypt1p under Heat-Shock by Characterizing a Temperature-Sensitive Mutant Yeast Strain, ypt1-G80D
Source: Int J Mol Sci. 2019 Jan 1;20(1):132. doi: 10.3390/ijms20010132 (PMC6337079; doi:10.3390/ijms20010132)
Supplement: Supplementary file 1 [file ijms-20-00132-s001.pdf]

International Journal of
Molecular Sciences

MDPI

Supplementary Materials:

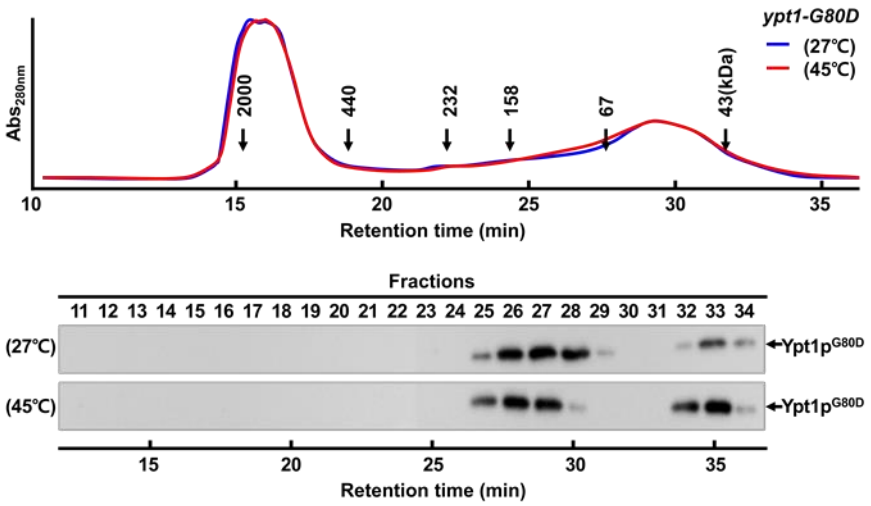

Figure S1. Heat-Shock does not Induce Changes in the Molecular State of Ypt1p^{G80D}. Mutant *ypt1-G80D* cells were grown in YPD medium (1 × 10^8 cells/ml) and incubated at 27°C or 45°C for 45 min. Subsequently, total cytosolic extracts of the cells were subjected to SEC analysis. The upper panel shows traces of the resolved protein peaks in the SEC analysis (upper image), and the lower panel shows immunoblot detection of Ypt1p^{G80D} in the corresponding fractions after SDS-PAGE. A 2.5 mg aliquot of total protein was applied to the SEC column, and 30 μl of each fraction was subjected to SDS-PAGE. Ypt1p^{G80D} was detected with a polyclonal anti-Ypt1p antibody.

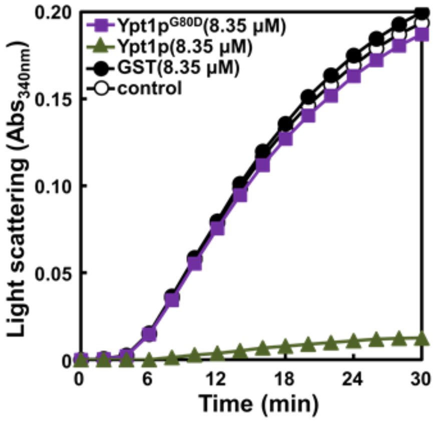

Figure S2. Ypt1p has Molecular Chaperone Activity, but Ypt1p^{G80D} does not. For the chaperone activity assay, light scattering was monitored at 340 nm over a 15 min incubation period. (C) Solutions of 1 μM CS alone (**-o-**) or with 8.35 μM GST (-●-), Ypt1p (-▲-), or Ypt1p^{G80D} (-■-) in 50 mM HEPES (pH 8.0) were incubated in a spectrophotometer cell at 43°C. Shown are representative data out of at least three independent experiments.

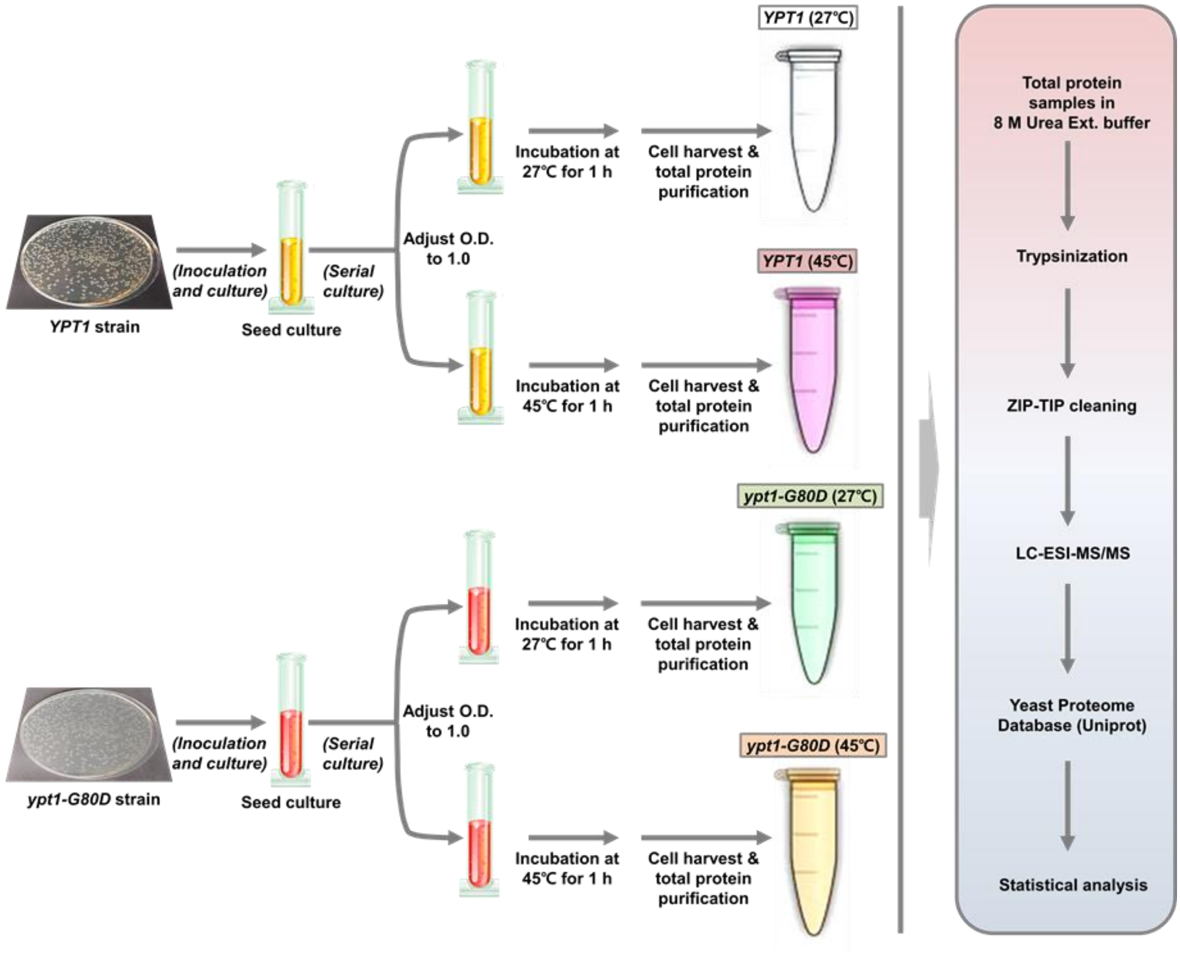

Figure S3. Experimental Workflow for the LC/MS Analysis Performed to Identify Putative Targets of Ypt1p Chaperone Activity.

Table S1. List of Heat-Shock-Induced Proteins that Accumulated in *YPT1* Cells but not y*pt1-G80D* Cells.

Rank	Accession	% Coverage	# PSMs	# Peptides	MW [kDa]	Protein name	Description
1	E7Q0L2	48.04	53	25	68.9	Ssa1p	Heat shock protein 70 family, ATP binding
2	E7Q5S7	61.06	37	18	54.8	ATP synthase subunit beta	ATPase alpha/beta chains family, ATP hydrolysis coupled proton transport
3	P00817	53.31	20	10	32.3	Inorganic pyrophosphatase	PPase family, phosphate-containing compound metabolic process
4	B3LNR6	68.55	18	10	30.4	Porin	Voltage-gated anion channel activity
5	E7NN07	27.03	16	12	84.5	Eft2p	GTPase activity, GTP binding
6	E7K9H0	34.85	14	8	50.8	Glucose-6-phosphate isomerase	GPI family, Gluconeogenesis
7	E7NEM0	23.5	12	8	61.3	Ssa3p	Heat shock protein 70 family, ATP binding
8	C8ZAJ3	39.6	8	6	27.9	Rhr2p	The haloacid dehydrogenase (HAD)-like hydrolase
9	E7KPS1	19.03	7	6	42.7	Om45p	None
10	A6ZYI0	19.37	6	3	24.2	ADK1 (Adenylate kinase)	Reversible transfer of the terminal phosphate group between ATP and AMP
11	C8Z3Y1	11.41	5	4	58.7	Ach1p	Acetyl-CoA metabolic process
12	B5VMN8	13.72	5	4	59.2	YKR097Wp-like protein	Gluconeogenesis, phosphoenolpyruvate carboxykinase (ATP) activity
13	E7KKX4	11.28	5	4	57.4	Thr4p	Cellular amino acid metabolic process, pyridoxal phosphate binding
14	E7QFS8	10.6	4	3	47.7	6-phosphogluconate dehydrogenase	Pentose-phosphate shunt, NADP binding
15	B3LTX2	3.16	3	2	114.3	Alpha-ketoglutarate dehydrogenase	Tricarboxylic acid cycle
16	B3RH70	36.07	3	3	24.3	Carboxypeptidase Y inhibitor	Carboxypeptidase activity
17	C8Z3T2	22.99	2	2	29.5	Prx1p	Antioxidant activity, peroxiredoxin activity
18	E7NGN8	12.8	2	2	29.1	Ses1p	Serine-tRNA ligase activity, ATP binding
19	P17536	17.59	2	2	23.5	Tropomyosin-1	Actin filament bundle assembly
20	B5VID1	9.36	2	2	42.1	YGL202Wp-like protein	Biosynthetic process, pyridoxal phosphate binding
21	E7KS25	16.14	2	2	28.0	Transaldolase	Pentose-phosphate shunt, carbohydrate degradation
22	E7LWQ4	4.07	2	2	64.1	Sdh1p	Electron transport chain, oxidoreductase activity
23	E7Q1Q6	27.5	2	2	17.0	Atp16p	ATP synthesis coupled proton transport, proton-transporting ATP synthase complex
24	P28241	11.11	2	2	39.7	IDH2 (Isocitric dehydrogenase 2)	Oxidoreductase, tricarboxylic acid cycle
25	A6ZLW7	1.62	1	1	84.5	Putative uncharacterized protein	Trehalose biosynthetic process, catalytic activity
26	B5VE05	15.34	1	1	17.9	YBR039Wp-like protein	ATP synthesis coupled proton transport, proton-transporting ATP synthase complex
27	B5VHZ0	7.72	1	1	37.2	YFL037Wp-like protein	GTPase activity, microtubule-based process
28	E7KMC6	19.09	1	1	12.5	Cytochrome b-c1 complex subunit 7	Mitochondrial electron transport, ubiquinol to cytochrome c
29	N1NWQ7	18.67	1	1	8.6	Pbi2p	Serine-type endopeptidase activity, negative regulation of catalytic activity
30	A6ZR65	2.43	1	1	122.5	KAP123 (Karyopherin beta 4)	Intracellular protein transport
31	A6ZW86	1.6	1	1	172.9	REV3 (DNA polymerase)	DNA-directed DNA polymerase activity
32	A6ZYB0	1.26	1	1	152.9	Putative uncharacterized protein	Cortical protein anchoring, phospholipid binding
33	A7A0P5	2.48	1	1	46.3	SOF1 (Conserved protein)	Contains WD repeat domains
34	A7A165	3.88	1	1	46.5	IDP2 (Isocitrate dehydrogenase)	Oxidoreductase, tricarboxylic acid cycle
35	B3LQM7	4.52	1	1	41.7	Branched chain a.a. aminotransferase	L-isoleucine transaminase activity
36	B5VDT3	11.7	1	1	21.1	YBL050Wp-like protein	Intracellular protein transport
37	B5VJ70	7.71	1	1	49.3	YGR124Wp-like protein	Asparagine biosynthetic process, asparagine synthase activity
38	B5VP02	5.63	1	1	45.2	YML128Cp-like protein	Contains Stress_response_Ish1 domain
39	B5VTE6	1.09	1	1	105.0	YPL016Wp-like protein	Contains ARID domain, DNA binding
40	C8ZII1	5.33	1	1	27.7	Rpl7bp	Ribosomal protein L30P family, Ribonucleoprotein
41	E7KJ59	8.56	1	1	33.7	Bts1p	FPP/GGPP synthase family, isoprenoid biosynthetic process
42	E7KR27	15.91	1	1	14.9	Tma19p	Contains Translationally controlled tumor protein (TCTP) domain
43	E7KW07	6.14	1	1	24.0	Enolase	Phosphopyruvate hydratase activity, carbohydrate degradation, glycolysis
44	E7NG58	7.41	1	1	24.0	Ypr1p	Oxidoreductase activity, contains Aldo_ket_red domain
45	E7NNH1	14.8	1	1	20.5	Erg10p	Transferase activity, transferring acyl groups other than amino-acyl groups
46	E7Q1X0	2.31	1	1	72.6	Mbp1p	Sequence-specific DNA binding transcription factor activity
47	E7Q465	4.51	1	1	54.1	Cys4p	Cystathionine beta-synthase activity, cysteine biosynthetic process from serine
48	G2WKH0	5.15	1	1	60.1	K7_Ade17bp	IMP cyclohydrolase activity, purine nucleotide biosynthetic process
49	P50263	20.25	1	1	8.9	SIP18	Phospholipid binding, cellular response to water deprivation

PSM, peptide-spectrum match. MW, molecular weight.
